# The influence of homocysteine and oxidative stress on pregnancy outcome

**Published:** 2012-03-05

**Authors:** O Micle, M Muresan, L Antal, F Bodog, A Bodog

**Affiliations:** University of Oradea, Medicine and Pharmacy Faculty, Preclinical II Department; University of Oradea, Medicine and Pharmacy Faculty, Surgery Department

**Keywords:** oxidative stress, hiperhomocysteinemia, pregnancy outcome

## Abstract

Oxidative stress in utero–placental tissues plays an important role in the development of placental-related diseases. Maternal hiperhomocysteinemia is associated with placental mediated diseases, such as preeclampsia, spontaneous abortion and placental abruption. The aim of our study is to appreciate the clinical usefulness of the dosage serum homocysteine and malondialdehyde, as an oxidative stress marker, in the pregnancies complicated with risk of abortion or preterm birth. The study was performed at the Obstetric Gynecology Clinical Hospital Oradea from December 2009 until April 2010. It included 18 patients with risk of abortion (group 1), 22 with preterm birth (group 2). The results were compared with a control group composed by 14 healthy pregnant women. Serum homocysteine level was measured by an enzymatic method, on the instrument Hitachi 912, Roche, reagent: Axis-Shield Enzymatic. For proving the oxidative stress we established the level of malondialdehyde using a method with thiobarbituric acid TBA (Kei Satoh 1978) and the level of ceruloplasmin with the Ravin method .Also AST, ALT,CRP, iron, uric acid, urea were assessed.

High level of homocysteine in both groups of study in comparison with the control group was found. The concentration of MDA was significantly higher in pregnancies complicated with risk of abortion and preterm birth compared to the control group (p=0.040, p=0.031). Considerable differences of ceruloplasmin concentration between group 1 and group 2 (p=0.045), and between group 2 and control group (p=0.034), was noticed but not any important differences between group 1 and control group (p=0.683). In women with risk of abortion or with preterm birth an oxidative stress and a hyperhomocysteinemia are present.

## Introduction

Placental-related disorders of pregnancy affect around a third of human pregnancies [**1**]. Changes in human lifestyle, such as delayed childbirth and diets customs, have increased the global incidence of placental-related disorders over the last decades [**2**].

Oxidative stress in utero–placental tissues plays an important role in the development of placental-related diseases.

Homocysteine, a sulfur-containing amino acid derives from the demethylation of methionine during DNA or/and RNA methylation. Increased homocysteine levels represent a risk factor in cardiovascular disease, osteoporosis, renal failure, diabetic microangiopathy, neuropsychiatric disorders [**3**].

Maternal hyperhomocysteinemia (Hcy) is frequently associated with placental mediated diseases, such as preeclampsia, spontaneous abortion and placental abruption [**4**]. Hyperhomocysteinemia induces the activations of NADPH oxidase and increases ROS [**5**].

For preventing hyperhomocysteinemia a lot of vitamins are prescribed during pregnancy. The folates belong to the vitamin B group and are involved in a large number of biochemical processes, including the metabolism of homocysteine. It is well known that folic acid supplementation impedes the neural tube defects, spontaneous abortion and recurrent pregnancy loss.

In spite of the acknowledged harmful effect of homocysteine in pregnancy, a lot of questions and problems remain unanswered and unsolved.

In our study we tried to estimate the clinical usefulness of the detection of the serum homocysteine and malondialdehyde in the placental related diseases and to evaluate the correlations between these markers in women with risk of abortion or preterm birth.

## Material and method

The study was performed at the Obstetric Gynecology Clinical Hospital Oradea from December 2009 until april 2010. It included 18 patients with risk of abortion (group 1), 22 with risk of preterm birth (group 2). The control group was matched with the studied groups for age, BMI (body mass index) and gestational age and consisted of 14 healthy pregnant women (group 3). All groups took oral vitamins supplementation which comprised 800 μg folic acid, 4.0 μg B12, 2.6 mg B6.

The diagnosis of risk of abortion is made for pregnancies less than 24 weeks of gestation, with uterine contractility and with local modifications based on changes in the Bishop score.

The risk of premature birth appears between 24 and 37 weeks of pregnancies, with painful uterine contractions (more than 3-4 contractions per hour) and local changes of the cervix, that are illustrated on a modified Bishop score.

For each of the patients enrolled we have a file with:

• age, residency, living conditions, profession, studies, toxics (smoking and caffeine ingestion)

• gestational age using last menstrual period and ultrasound confirmation

• waist, weight, calculated BMI

• blood pressure and sitting pulse

• a physical examination and a gynecological one

• an obstetrical ultrasound investigation for fetal biometry, viability, gestational age, biparietal diameter, head circumference, abdominal circumference, femur length. 

• a Doppler examination on uterine artery, umbilical artery and medial cerebral artery

All groups were enrolled in the study after having been informed and subscribed a written consent. The study was approved by the institutional ethical committee.

For each of the patients blood samples were collected from antecubital veins of the subjects in supine position before any medication, with all aseptic precautions after an overnight fasting. Samples were centrifuged and serum was separated and stored at -35°C until analysis.

Serum homocysteine level was measured by an enzymatic method, on instrument Hitachi 912, Roche, reagent: Axis-Shield Enzymatic Homocysteine Assay Cat No.FHER100. For proving the oxidative stress we established the level of malondialdehyde using a method with thiobarbituric acid TBA (16) and the level of ceruloplasmin with the Ravin method. In the same time the level of AST/GOT, ALT/GPT, CRP(C reactive protein), iron, uric acid and urea was tested. Data are expressed as mean ± SD (standard deviation). The significant differences was assessed using the Student`s test. P value < 0.05 was considered statistically significant. The correlation between parameters was estimated by Pearson`s correlation coefficient (r).

## Results

There were no significant difference between gestational age, arterial pressure and body mass index (BMI) among the studied pregnant women and the control group (**Table 1**). The mean levels of serum homocysteine, MDA, ceruloplasmin, AST, ALT, CRP, iron, uric acid, urea are shown in table no.1.

**Table 1:** Characteristics of study and control group

	Group 1	Group 2	Control group
N	18	22	14
Age (years)	27,8±2,6	28,4±3,0	29,2±4,3
Mean arterial pressure (mmHg)	112/70	113/72	116/73
BMI(body mass index)	23,93	28,44	27,72
AST/GOT(U/L)	16,66±1,32	14,47±2,09	13,23±1,78
ALT/GPT(U/L)	10,90±1,58	10,30±2,26	10,98±2,18
Ureea (g/L)	0,12±0,05	0,12±0,02	0,17±0,02
Uric acid (mg/dL)	2,59±0,35	2,87±0,58	3,08±0,64
Iron (μg/dL)	87,13±22,45	70,08±21,31	65,36±10,63
CRP (mg/L)	4,95±2,93	3,20±2,27	2,07±0,48
Homocysteine (μmol/L)	5,33±0,66	5,70±0,72	4,91±0,67
Malondialdehyda (nmol/ml)	2,59±0,38	2,82±0,41	2,46±0,35
Ceruloplasmin (mg/dl)	48,63±6,08	44,76±4,99	49,55±8,15
group 1= risk of abortion, group 2= risk of preterm birth, group 3= healthy pregnant women			

**Fig. 1:**
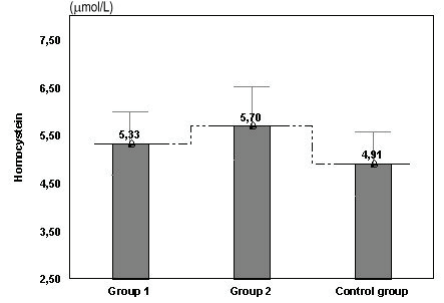
Values of homocysteine in women with risk of abortion and risk of preterm birth compared with the control group

In the first group of women with risk of abortion homocysteine concentration was higher compared to the control group (from 4.91μmol/l in control group to5.33 μmol/l in group 1). The group of women with risk of preterm birth had also an elevated level of the seric homocysteine (5.70μmol/l and 4.91μmol/l in the reference group). Homocysteine concentration between group 1 and group 2 (p=0.05), between group 1 and control group (p=0.041), and between group 2 and control group (p=0.022) is statistically remarkable.

We have obtained higher values for pregnancies with risk of preterm birth than for those with risk of abortion (**Fig. 1**).

**Fig. 2:**
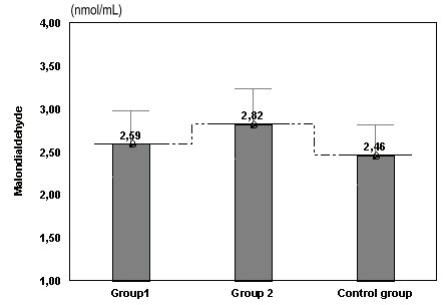
Values of MDA in women with risk of abortion or preterm birth compared with the control group

The mean values for MDA was 2.82 nmol/ml in pregnant women with risk of preterm birth, 2.59 nmol/ml in women with risk of abortion and the smallest value 2,46 nmol/ml in the control group. There are no important differences between group 1 and control group (p>0.05), but the difference is significant between group 2 and group 1, respectively the control group (p=0.040, p=0.031).

We obtained the smallest value for the control group and the highest one in the group of women with risk of preterm birth, but without statistical importance. There is a considerable variation between the women with risk of preterm birth, with higher MDA value than those in the group of women with risk of abortion (**Fig. 2**).

**Fig. 3:**
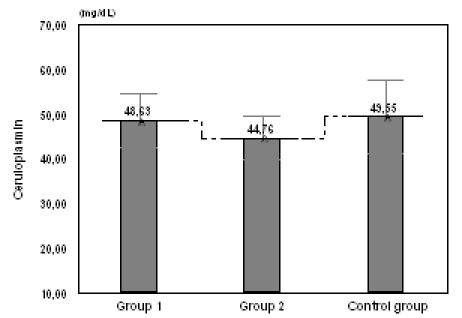
Values of ceruloplasmin in women with risk of abortion or risk of preterm birth compared with the control group

We obtained important differences of ceruloplasmin between group 1 and group 2 (p=0.045), and between group 2 and the control group (p=0.034), and no significant differences between group 1 and the control group (p=0.683) (**Fig. 3**).

**Fig. 4:**
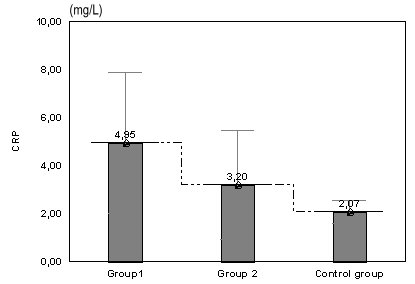
Values of CRP in women with risk of abortion and preterm birth compared with the control group

The mean levels of CRP is higher in group in comparison with the group 2 (p=0.042), and also group 2 likened with the control group (p=0.030).

**Fig. 5:**
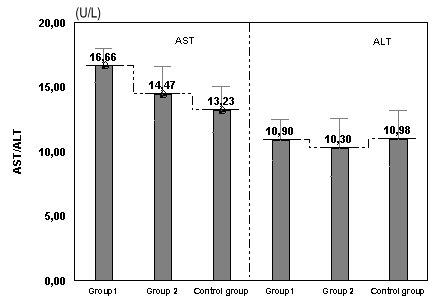
Values of AST (GOT)/ ALT (GPT) in women with risk of abortion and preterm birth compared with the control group

Variations without importance of urea and uric acid are present between group1, group 2 and the control group (p>0.05) (**Table 1**). The level of ALT/GPT is similar in all the three groups (p>0.05). Significant differences of the concentration of AST/GOT between group 1 and group 2 (p=0.011), between group 1 and the control group (p<0.001), appeared but not between group 2 and the control group (p= 0.593) (**Fig. 5**).

**Fig. 6:**
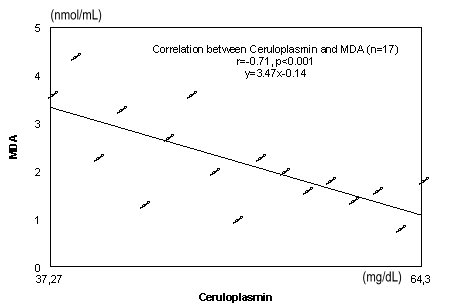
Correlation between ceruloplasmin and MDA

Significant negative correlations were observed between serum concentration of ceruloplasmin and MDA r=-0.71, p<0.001(**Fig. 6**).

**Fig. 7:**
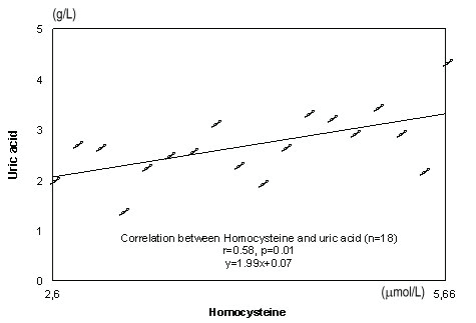
A significant positive correlation can be seen between serum concentration of homocysteine and uric acid r=0.58, p=0.01 in Figure 7

**Fig. 8:**
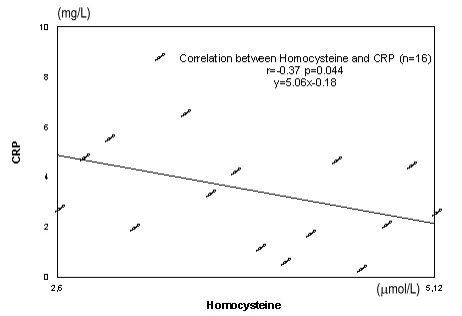
Correlation between homocysteine and CRP

Significant negative correlation were observed between serum concentration of homocysteine and CRP r=-0.37, p=0.044 (**Fig. 8**).

## Discussion

Due to the perturbations in methionine metabolism, hyperhomocysteinemia during pregnancy is implicated in adverse outcomes such as neural tube defects, preeclampsia, spontaneous abortion, and premature delivery [**6**].

In our study serum homocysteine concentration was significantly high in both groups of women with risk of abortion and with the risk of preterm birth compared to the control. The level of homocysteine was considerably increased in the group of women with risk of preterm birth compared to the reference group (p=0.022) and also in the group of women with risk of abortion.

The serum concentration of homocysteine was higher in the group 2 in comparison with group 1 (p=0.05).Our results were similar to those obtained by other researchers [**7,8**].

All the patients received a vitamin supplementation, having role in the prevention of homocysteinemia and of premature vascular disease in pregnant women. Early damage to decidual or corionic vessels may cause abnormal implantation of the blastocyst. On the other hand some papers point out that the disturbed folate metabolism is not an apparent risk factor for spontaneous first-trimester pregnancy loss [**9**]. More specific studies regarding type of vitamin and its usefulness need to be conducted.

A significant positive correlation can be seen between serum concentration of homocysteine and uric acid. The explanation of this is that homocysteine induces endothelial cells lesions and release purine nucleotides. Catabolism of the purine nucleotides leads finally to the production of uric acid. Uric acid is one of the plasma antioxidants which protect the cells against increased ROS activity.

Significant negative correlations were observed between serum concentration of homocysteine and CRP.

Homocysteine is a potent excitatory neurotransmitter that binds to the N-methyl-D-aspartate (NMDA) receptor and also leads to oxidative stress, cytoplasmic calcium influx, cellular apoptosis, and endothelial dysfunction [**10**].

There are a lot of researches which put in evidence that oxidative stress in utero–placental tissues with an important role in the development of placental-related diseases. 

Ezashi et al. 2005 [**11**], pointed out that the hypoxia is necessary to maintain stem cells in a fully pluripotent state. Physiological level of reactive oxygen species regulates the transcription factors [**12**]. In normal pregnancies, the earliest stages of the fetus development take place in a physiological hypoxia. This protects the developing fetus against the deleterious and teratogenic effects of reactive oxygen species (ROS).

In women with risk of abortion, the development of the placento–decidual interface is severely impaired leading to early and widespread onset of maternal blood flow and major oxidative damage. This mechanism is common to all miscarriages, especially in the first trimester of pregnancy depending also on the etiology [**1**].

The levels of lipid peroxides are too increased in the villous and decidual tissues of women undergoing early pregnancy loss [**13,14**].

In our study there are no significant differences of MDA between group1 and control group (p>0.05), but they appears between group 2 and reference group (p=0.031) and also significant between the studied groups.

Baxter et al. [**15**] have shown that women with naturally higher levels of antioxidant enzymes are less likely to miscarry. The concentration of ceruloplasmin in the group of women with risk of abortion was considerably higher than in the group of women with risk of preterm birth (p=0.045), in comparison with healthy pregnant women (p=0.034). Similar values was noticed in the group 1 and the control group (p=0.683).

The enzyme AST/GOT catalyzes the transfer of the amino group of the aspartic acid to α ketoglutaric acid, forming glutaminic and oxaloacetic acid. After extensive tissue destruction AST/GOT and ALT/GPT are liberated into the serum. The elevated serum level of AST/GOT in the group of women with risk of abortion indicates an utero-placental damage.

C-reactive protein (CRP) rises in the early phase of inflamation. The higher value of CRP associated with increased concentration of AST/GOT, oxidative stress and hiperhomocysteinemia represents a signal of severe utero-placental disturbance.

These results offer arguments for an early prophylaxis with antioxidant vitamins which decreases homocysteine levels, oxidant activity and in the same time can restore the endothelial malfunction.

## Conclusions

1. Serum homocysteine concentration was significantly higher in both groups of women with abortion risk and of preterm birth compared with the control group.

2. A significant positive correlation can be seen between serum concentration of homocysteine and uric acid demonstrating indirectly the harmful action of hiperhomocysteinemia upon the endothelium.

3. In both studied groups there was an augmentation of MDA in comparison with the control group.

4. The rise of AST/GOT and of CRP in women with risk of abortion constitutes an indicator of utero-placental disorders.

**Acknowledgements**

This paper is supported by the authors.
